# Associations between ACE-Inhibitors, Angiotensin Receptor Blockers, and Lean Body Mass in Community Dwelling Older Women

**DOI:** 10.1155/2018/8491092

**Published:** 2018-02-19

**Authors:** Jennifer W. Bea, Sylvia Wassertheil-Smoller, Betsy C. Wertheim, Yann Klimentidis, Zhao Chen, Oleg Zaslavsky, Todd M. Manini, Catherine R. Womack, Candyce H. Kroenke, Andrea Z. LaCroix, Cynthia A. Thomson

**Affiliations:** ^1^University of Arizona Cancer Center, 1515 N. Campbell Ave, P.O. Box 245024, Tucson, AZ 85724-0524, USA; ^2^Department of Epidemiology and Population Health, Albert Einstein College of Medicine of Yeshiva University, 1300 Morris Park Avenue, Belfer Building, Room 1308B, Bronx, NY 10461, USA; ^3^Department of Epidemiology and Biostatistics, Mel and Enid Zuckerman College of Public Health, University of Arizona, 1295 N. Martin, P.O. Box 245211, Drachman Hall A238, Tucson, AZ 85724, USA; ^4^Department of Biobehavioral Nursing and Health Systems, University of Washington, P.O Box 357266, 1959 NE Pacific Ave., Seattle, WA 98195-7266, USA; ^5^Department of Aging and Geriatric Research, University of Florida, P.O. Box 100107, Gainesville, FL 32610, USA; ^6^Department of Medicine, University of Tennessee, 956 Court Avenue, Memphis, TN 38163, USA; ^7^Kaiser Permanente Northern California, Division of Research, 2000 Broadway, Oakland, CA 94612, USA; ^8^Department of Family Medicine and Public Health, University of California, 9500 Gilman Drive No. 0725, San Diego, La Jolla, CA 92093, USA; ^9^Department of Health Promotion Sciences, Mel and Enid Zuckerman College of Public Health, University of Arizona, 1295 N. Martin, P.O. Box 245209, Drachman Hall A260, Tucson, AZ 85724, USA

## Abstract

Studies suggest that ACE-inhibitors (ACE-I) and angiotensin receptor blockers (ARBs) may preserve skeletal muscle with aging. We evaluated longitudinal differences in lean body mass (LBM) among women diagnosed with hypertension and classified as ACE-I/ARB users and nonusers among Women's Health Initiative participants that received dual energy X-ray absorptiometry scans to estimate body composition (*n*=10,635) at baseline and at years 3 and 6 of follow-up. Of those, 2642 were treated for hypertension at baseline. Multivariate linear regression models, adjusted for relevant demographics, behaviors, and medications, assessed ACE-I/ARB use/nonuse and LBM associations at baseline, as well as change in LBM over 3 and 6 years. Although BMI did not differ by ACE-I/ARB use, LBM (%) was significantly higher in ACE-I/ARB users versus nonusers at baseline (52.2% versus 51.3%, resp., *p*=0.001). There was no association between ACE-I/ARB usage and change in LBM over time. Reasons for higher LBM with ACE-I/ARB use cross sectionally, but not longitundinally, are unclear and may reflect a threshold effect of these medications on LBM that is attenuated over time. Nevertheless, ACE-I/ARB use does not appear to negatively impact LBM in the long term.

## 1. Introduction

Sarcopenia, defined as a progressive loss of skeletal muscle mass, is a significant public health concern linked to diminished quality of life, disability, and even mortality in older adults [1, 2]. Investigating strategies to increase muscle mass or minimize losses in older adults is important and a bourgeoning area of research [1, 2]. Correspondingly, within older populations, antihypertensive medications, such as ACE-inhibitors (ACE-I) and angiotensin receptor blockers (ARBs), are regularly prescribed to those at risk for or diagnosed with hypertension and cardiovascular diseases (CVDs) [3]. Interestingly, these medications target angiotensin II, a protein upregulated in hypertension and CVD, which has been associated with a phenomenon called cardiac cachexia, in which the skeletal muscle mass is lost more rapidly than would be expected under normal aging conditions [4]. Thus, ACE-I and ARBs, respectively, may help to preserve lean body mass (LBM) among older adults. Studies suggest that these medications not only target smooth muscle relaxation, aiding in blood pressure control, but also have a direct positive effect on skeletal muscle [2, 4, 5].

The extent of ACE-I and ARB mechanisms of action on muscle is not fully understood; the potential favorable effects of these medications on muscle mass and function could inform on the best options for blood pressure control, particularly for older individuals [5]. In fact, the Health ABC study, including >2400 elderly males and females, found that larger lower extremity muscle mass was associated with ACE-I use [6]. Meanwhile, functional studies related to skeletal muscle performance have yielded mixed results. For example, the Women's Health Initiative (WHI) found no associations between ACE-Is and frailty, physical performance, and strength with 3 and 6 years follow-up, respectively [7, 8]. Similarly, Health ABC found no association between ACE-I and risk of mobility limitations over 6.5 years [9]. In contrast, the Women's Healthy Aging Study found positive associations between ACE-I, muscle strength, and walking speed at 3 years, even after adjusting for cardiovascular events [10]. Unfortunately, these prior analyses were limited to those over the age of 65 years, and none of them directly evaluated LBM prospectively. However, hypertension, commonly treated with ACE-I/ARBs, is present in 34% of US adults aged 45–64 years [11].

Fortunately, a subset of WHI participants (*n*=10,635) completed repeated body composition over six years. Of those with DXA scans, 2642 self-reported being currently treated for hypertension. The aim of the present analysis was to evaluate baseline differences in LBM among ACE-I/ARB users and nonusers and to determine if longitudinal changes in LBM are associated with ACE-I/ARB use among postmenopausal women. We hypothesized that taking antihypertensive medications, specifically ACE-I and ARBs, would be associated with higher LBM and preservation of LBM with aging.

## 2. Methods

### 2.1. Study Population

Between 1993 and 1998, healthy postmenopausal women aged between 50 and 79 years were enrolled in the WHI clinical trials and observational study at 40 clinical centers across the U.S. (*n*=161,808) [12, 13]. A subcohort consented to complete body composition measurements at the WHI clinical centers in Pittsburgh, PA; Birmingham, AL; and Tucson-Phoenix, AZ (*n*=11,020) [14]. All participants with body composition at baseline and for whom medication information could be ascertained were included in the analysis (*n*=10,635); of those, 2642 were treated for hypertension at baseline. The protocol and consent forms were approved by the institutional review board at each site, and all participants provided written informed consent. Study design and participant characteristics have been described in detail elsewhere [12, 13, 15].

### 2.2. Body Composition

Total and regional body composition was determined by performing total body dual energy X-ray absorptiometry (DXA) scans (QDR2000, 2000+, or 4500W; Hologic Inc, Bedford, MA) at baseline and at years 3 and 6. Percent total body fat [%TBF = TBF (kg)/weight (kg)], LBM [%LBM = LBM (kg)/weight (kg)], and appendicular lean mass [aLM% = LBM_arms_ + LBM_legs_ (kg)/weight_arms_ + weight_legs_ (kg)] were computed. DXA-derived fat and lean mass have been validated against gold standard, magnetic resonance imaging, within a subset of this study population (*n*=101; WHI MRI precision error < 1%; fat, Pearson rho = 0.99; lean, Pearson rho = 0.94, *p* < 0.001) [16].

The WHI DXA quality assurance program has been well described previously. It included standard positioning and analysis protocols; certification of technicians; local daily and weekly phantom scans; circulating Hologic calibration phantoms; and machine and technician performance monitoring by review of phantom and problematic scans, as well as random sampling [17].

### 2.3. Medications

Participants brought all current medications to their baseline and year 3 visit at the local WHI clinics. All medications were assigned drug codes in the Medi-Span software database (First DataBank, Inc., San Bruno, CA). The antihypertensive medications of interest in this analysis, ACE-I and ARBs, were coded according to therapeutic classes as follows: ACE Inhibitors; Angiotensin II Receptor Antagonist; ACE Inhibitors and Calcium Blockers; ACE Inhibitors and Thiazide/Thiazide-Like; and Angiotensin II Receptor Antagonist and Thiazides. For baseline analyses, binary categorization of users and nonusers of ACE-I or ARB was used. For follow-up analyses, the following categories were created: nonuse, no use at baseline or year 3; intermediate use, use at either time point; use, use at both time points.

### 2.4. Covariates

Potential covariates were initially identified from published literature evaluating body composition and medications. Self-report questionnaires were used to determine demographic characteristics, health history, and lifestyle behaviors, such as age, neighborhood socioeconomic status (NSES) [18], race/ethnicity, dietary energy consumption (kcal/day) [19] and quality (Healthy Eating Index, HEI 2005) [20], physical activity (MET-hr/wk) [21], physical function [22], smoking (pack-years), and alcohol use. Polypharmacy categories (0–4 or ≥5 medications) were created utilizing all reported medications. Weight (kg) and height (cm) were measured during clinic visits on a balance-beam scale and wall-mounted stadiometer, respectively. Body mass index (BMI) was calculated as weight (kg)/height (m)^2^.

### 2.5. Statistical Analysis

Baseline characteristics were compared between ACE-I/ARB users and nonusers by *t*-test for continuous variables and chi-square tests for categorical variables. Within the full DXA cohort (*n*=10,635), key potential confounding variables were found to be clinically and statistically significantly different between ACE-I/ARB users (*n*=944) and nonusers (*n*=9691). ACE-I/ARB users were significantly older (2 years), physically larger (2.0 kg/m^2^), and more racially/ethnically diverse and reported less education, poorer health, lower alcohol consumption, lower hormone therapy use, lower physical activity, lower energy intake, and greater use of other CVD-related medications than nonusers (*p* ≤ 0.005; *data not presented*). Therefore, the present analysis is primarily focused on those reporting being treated for hypertension at baseline (*n*=2642), to better evaluate the differences between ACE-I/ARB users (*n*=782) and nonusers (*n*=1860) rather than the differences between those treated for hypertension and those without hypertension. All analyses were repeated in the full cohort for comparison.

The cross-sectional association between ACE-I/ARB use and LBM at baseline was assessed by linear regression. Absolute and relative *changes* in LBM from baseline to year 3 or year 6 based on exposure to ACE-I/ARB during the first three years of the study period were evaluated using multivariate-adjusted linear regression models and the ACE-I/ARB use classifications above. Covariates were scanner serial number; clinical trial arm; self-reported age, race/ethnicity, smoking, physical activity, history of diabetes treatment, history of arthritis, cancer, CVD, depressive symptoms, HRT use (never, past, and current), general health (excellent, very good, good, or fair/poor), physical function (dichotomized as <90 or ≥90 points), dietary energy, dietary protein, and HEI; polypharmacy; SES; and clinic measured systolic and diastolic blood pressure and height at baseline. Models evaluating change in LBM were further adjusted for baseline lean mass measure and year 3 physical activity. The effects of *new* use of either drug at year 3 on change in LBM and aLM between years 3 and 6 were explored in similar models. New users were defined by ACE-I/ARB use reported at year 3 but not at baseline (*n*=174), which was compared to no ACE-I/ARB use reported at baseline nor year 3 (non-users, *n*=837). Due to the high levels of missing data among covariates, multiple imputation with chained equations was employed to fill in the gaps [23]. The imputation model used all variables listed above as covariates. Twenty complete datasets were created, analyzed, and combined. All statistical analyses were conducted using Stata 15.1 (StataCorp, College Station, TX), and significance was set at *p* < 0.05.

## 3. Results

Among those being treated for hypertension (*n*=2642), there were significant differences between ACE-I/ARB users (*n*=782) and nonusers (*n*=1860) in terms of age, SES, race/ethnicity, smoking status, or hormone therapy use (Table 1). Users had slightly higher systolic, but not diastolic, blood pressure and were more likely to be treated for diabetes, prescribed a greater number of medications (polypharmacy), and reported lower general health. Diet quality was higher among users. Absolute body size and composition were not significantly different at baseline between users and nonusers; however, TBF (%) was 0.8% higher and LBM (%) was correspondingly lower among nonusers at baseline.

Total body lean mass (kg) was significantly greater among ACE-I/ARB users compared to nonusers in fully adjusted models at baseline (Table 2; lean mass, kg: *β*: 0.42; standard error (SE): 0.20; *p* < 0.04). The absolute lean mass association with ACE-I/ARB use stratified by BMI (normal weight (18.5–24.9 kg/m^2^), overweight (25.0–29.9 kg/m^2^), and obese (≥30 kg/m^2^)) remained significant for normal weight (0.77 (0.28); *p*=0.007) and overweight (0.67 (0.22); *p*=0.002) categories, but not for obese women (0.14 (0.28); *p*=0.621). Percent LBM and aLM were also significantly greater among ACE-I/ARB users than nonusers (*β*: 0.76, SE: 0.26 and *β*: 1.08, SE: 0.28, resp.; both *p* < 0.01).

Baseline ACE-I/ARB use was not significantly associated with total LBM or aLM change in between baseline and the year 3 visit. However, a substantial proportion (21%) of participants did not have data available at year 3, and these women were different from those with follow-up data with regard to several baseline characteristics. Women lacking follow-up data were significantly older with lower NSES, lower physical activity, higher systolic blood pressure, higher BMI, and lower HEI than women with follow-up data. Additionally, those without follow-up were more likely than those with follow-up data to be black or Hispanic, smoke currently or previously, take 5+ prescription medications, and have diabetes, arthritis, or depressive symptoms. Furthermore, women lacking follow-up data had significantly worse physical functioning and general health than women with follow-up data. There were no significant differences between these two groups of women with regard to personal history of cancer or cardiovascular disease or dietary energy or protein (data not shown).

Similarly, change in total LBM or aLM was not different between ACE-I/ARB use categories over 3 years and 6 years (Table 3). All analyses related to *change* in lean mass comparing ACE-I/ARB users to nonusers, regardless of hypertensive status, were repeated using the full DXA cohort (*n*=10,635), and results remained null (*data not presented*).

## 4. Discussion

Within the largest and most diverse prospective study of postmenopausal women with body composition information, we found ACE-I/ARB use to be significantly associated with higher LBM in cross-sectional analyses. However, ACE-I/ARB use among hypertensives was not associated with change in LBM or aLM longitudinally. The higher lean mass with ACE-I/ARB use that was demonstrated cross sectionally, but not longitudinally, herein could reflect a threshold effect of these medications on LBM that is attenuated over time. The fact that ACE-I/ARB use did not negatively impact LBM long term is important clinically in that it does not deter clinicians from prescribing these medications long term.

Reducing angiotenin II or its activity via ACE-I and ARBs, respectively, was hypothesized to preserve LBM in postmenopausal women, a population that losses significant LBM annually through the end of life [24]. Health ABC findings of greater lower extremity LBM in older persons using ACE-I cross sectionally, similar to the cross-sectional differences in ACE-I/ARB users versus nonusers herein, suggest LBM as a possible explanation of the benefits of ACE/ARB use in wasting syndromes [6]. However, no longitudinal effects on LBM were demonstrated within the WHI. Results from a previous analysis of 25% random sample of WHI clinical trial participants aged ≥65 demonstrated no difference in mean annual change in function over time among hypertensives taking ACE-I compared to nonusers [8]. Similar null effects on grip strength were reported for the Hertfordshire Cohort Study (*n*=639, 50% female, aged 65 yrs) for ACE-I use [25].

The robust measure of body composition, detailed characterization of the cohort, broader age range, diversity, and large sample size were strengths of the present analysis. Similar results across LBM and aLM indicate that health conditions that may influence LBM measures by DXA, such as organ enlargement with serious health conditions, did not significantly influence results.

Accounting for body size was important, with height being a major confounder, significantly increasing the point estimate. Limiting to hypertensive individuals in the primary analyses and separately evaluating results by BMI strata further minimized the potential for cross-sectional associations between LBM and ACE-I/ARB to be due to larger body sizes typically associated with hypertension and CVD. Lifestyle factors (e.g., diet and physical activity) were included in the models to minimize behavioral confounding. It is important to note that we adjusted for other important covariates such as race/ethnicity, blood pressure, diabetes treatment, and polypharmacy given that clinical guidelines have begun to differentiate treatment strategies based on various characteristics such as hypertensive stage at presentation, race, diabetes, and other comorbidities [26].

### 4.1. Limitations

The present analysis cannot be applied to men or younger women. Minimal overlap between DXA and physical function subcohorts precluded concomitant evaluation. Comparisons between drug classes and monotherapy versus combination therapy (i.e., ACE-I combined with thiazide) were limited by power and prescribing patterns of the time [27], similar to other large studies [6]. Patients prescribed an ACE-I commonly switch to an ARB and vice versa depending on side effects and may do so several times during the course of treatment. Therefore, although we recognize that the medications may have different effects, beyond the hemodynamic effects for which they are prescribed, we could not adequately account for crossovers given the periodicity of medication monitoring within WHI, and we examined both antihypertensive medications in combination. In addition, we did not have information on the doses prescribed or taken, prohibiting examination between dose and LBM. Though multiple imputation was employed to account for missing covariates, differences between participants lost to follow-up and those remaining in the analysis may have introduced some bias.

Nevertheless, ACE-I and ARB medications continue to be routinely prescribed early in the medical management of CVD, heart failure, and hypertension, particularly if the patient presents with stage 2 hypertension [26, 28–30]. We expect that new medications, including those that affect the Renin–angiotensin–aldosterone system (e.g., angiotensin II receptor blocker neprilysin inhibitors) [31] will be increasingly incorporated in the management of hypertension. We could not examine these newer medications. Comparisons between medications in relation to their effects on body composition should continue and will be facilitated by the advent of an ICD-10 Code specific to sarcopenia (M62.84, as of Oct. 1, 2016).

In conclusion, our results support the use of ACE-I/ARB use without concern for deleterious changes in LBM among hypertensives. New cohorts are needed to determine if there are differences in the effects on LBM between the two common antihypertensive medications examined here, as well as newer medications.

## Figures and Tables

**Table 1:** Baseline characteristics of hypertensive women in the Women's Health Initiative DXA Cohort by ACE-I/ARB use^∗^^†^.

Characteristic: mean ± SD or *n*%	Total *n*=2642	Nonusers *n*=1860	Users *n*=782	*p*
Age (years)	65.1 ± 7.2	65.0 ± 7.2	65.1 ± 7.2	0.666
NSES	69.2 ± 10.3	69.0 ± 10.5	69.5 ± 9.8	0.283
*Race/ethnicity*				0.112
Non-Hispanic white	1736 (65.7)	1205 (64.8)	646 (68.4)	
Black	737 (27.9)	543 (29.2)	194 (24.8)	
Hispanic	116 (4.39)	77 (4.14)	39 (4.99)	
Other or unknown	53 (2.01)	35 (1.88)	18 (2.30)	
*Smoking status*				0.744
Never	1471 (56.6)	1029 (56.4)	442 (57.0)	
Former	961 (37.0)	673 (36.9)	288 (37.1)	
Current	169 (6.50)	123 (6.74)	46 (5.93)	
Physical activity (MET-hr/wk)	9.44 ± 12.3	9.36 ± 11.8	9.64 ± 13.5	0.593
*Blood pressure*				
Systolic (mmHg)	138.2 ± 18.4	137.7 ± 18.1	139.5 ± 19.2	0.021
Diastolic (mmHg)	77.0 ± 9.7	77.1 ± 9.7	76.8 ± 9.7	0.390
*Body size*				
Height (cm)	161.2 ± 6.5	161.3 ± 6.6	160.8 ± 6.3	0.080
Weight (kg)	78.8 ± 17.7	79.1 ± 18.3	78.0 ± 16.2	0.138
BMI (kg/m^2^)	30.2 ± 6.3	30.2 ± 6.4	30.1 ± 5.9	0.668
Lean mass (kg)	39.1 ± 6.0	39.0 ± 6.1	39.3 ± 5.9	0.316
Lean mass (%)	51.5 ± 6.6	51.3 ± 6.7	52.1 ± 6.5	0.005
Fat mass (%)	45.7 ± 6.9	45.9 ± 7.0	45.1 ± 6.7	0.009
Appendicular lean mass (%)	45.7 ± 6.8	45.3 ± 6.9	46.5 ± 6.7	<0.001
*Medical history*				
Diabetes treatment	327 (12.4)	189 (10.2)	138 (17.7)	<0.001
Polypharmacy (≥5)	980 (37.1)	623 (33.5)	357 (45.7)	<0.001
Depressive symptoms	375 (14.6)	271 (15.0)	104 (13.7)	0.399
Arthritis	1653 (62.9)	1163 (62.8)	490 (62.9)	0.973
Cancer	216 (8.22)	151 (8.15)	65 (8.37)	0.857
Cardiovascular disease	726 (27.9)	527 (28.8)	199 (25.7)	0.112
*General health*				0.001
Excellent	132 (5.02)	105 (5.66)	27 (3.48)	
Very good	661 (25.1)	480 (25.9)	181 (23.4)	
Good	1231 (46.8)	876 (47.3)	355 (45.8)	
Fair/poor	605 (23.0)	393 (21.2)	212 (27.4)	
Physical function construct <90	1808 (70.1)	1276 (70.2)	532 (69.9)	0.888
*Hormone replacement therapy*				0.686
Never used	1288 (48.8)	914 (49.2)	374 (47.8)	
Past use	465 (17.6)	320 (17.2)	145 (18.5)	
Current use	888 (33.6)	625 (33.6)	263 (33.6)	
*Diet*				
Energy intake (kcal/d)	1656 ± 724	1681 ± 735	1595 ± 694	0.006
Protein intake (%)	16.6 ± 3.3	16.5 ± 3.3	16.8 ± 3.3	0.035
Healthy Eating Index	65.4 ± 11.3	65.1 ± 11.4	66.2 ± 11.0	0.023

^∗^ACE-inhibitor, ACE-I; angiotensin receptor blocker, ARB; cardiovascular disease, body mass index, BMI; Healthy Eating Index, HEI; hormone replacement therapy, HRT; neighborhood socioeconomic status, NSES; standard deviation, SD; ^†^missing data: NSES 42; smoking 41; physical activity 9; blood pressure (systolic/diastolic) 2/3; height 15; weight 4; BMI 19; diabetes 6; depression 72; arthritis 12; cancer 13; CVD 36; general health 13; physical function 63; HRT 1; diet (energy, protein, HEI) 125.

**Table 2:** Association between baseline ACE-I/ARB use and measures of lean mass using linear regression in hypertensive women from the Women's Health Initiative^∗^.

Outcome	Baseline lean mass *β* (SE) *n*=2642		Change in lean mass (baseline to year 3) *β* (SE) *n*=2095	
	Model 1^†^	Model 2^‡^	Model 1^†^	Model 2^‡^
*Lean mass (kg)*				
Nonuser	Ref.	Ref.	Ref.	Ref.
User	0.31 (0.25)	0.42 (0.20)	0.06 (0.09)	0.14 (0.09)
*p* value	0.212	0.038	0.481	0.137
*Lean mass (%)*				
Nonuser	Ref.	Ref.	Ref.	Ref.
User	0.73 (0.28)	0.76 (0.26)	−0.28 (0.14)	−0.21 (0.15)
*p* value	0.009	0.004	0.055	0.141
*Appendicular lean mass (%)*				
Nonuser	Ref.	Ref.	Ref.	Ref.
User	1.16 (0.29)	1.08 (0.28)	−0.14 (0.15)	−0.12 (0.16)
*p* value	<0.001	<0.001	0.358	0.452

^∗^ACE-inhibitor, ACE-I; angiotensin receptor blocker, ARB; ^†^Model 1 adjusted for scanner serial number. The change model is also adjusted for the baseline lean mass measure; ^‡^Model 2 further adjusted for age, neighborhood socioeconomic status, race/ethnicity, smoking, physical activity, systolic blood pressure, diastolic blood pressure, height, diabetes, polypharmacy, depressive symptoms, arthritis, cancer, cardiovascular disease, hormone replacement therapy use, general health, physical function, dietary energy, dietary protein, and healthy eating index. The change model is also adjusted for physical activity at year 3 and clinical trial arm(s). Multiple imputation was used to fill in missing values for baseline covariates (see Methods for details and Table 1 footnote for missing data frequencies); however, participants missing medication use at year 3 were excluded from this analysis (*n*=35).

**Table 3:** Association between ACE-I/ARB use over time and change in lean mass in hypertensive women from the Women's Health Initiative^∗^.

	Baseline to year 3 *β* (SE); *p* *n*=2060		Baseline to year 6 *β* (SE); *p* *n*=1687	
	Model 1^†^	Model 2^‡^	Model 1^†^	Model 2^‡^
*Lean mass (kg)*				
Nonuser	Ref.	Ref.	Ref.	Ref.
Intermediate user	0.06 (0.10); 0.54	0.10 (0.10); 0.34	0.08 (0.15); 0.59	0.08 (0.15); 0.58
User	0.07 (0.11); 0.52	0.18 (0.11); 0.12	0.23 (0.16); 0.16	0.29 (0.16); 0.08
*Lean mass (%)*				
Nonuser	Ref.	Ref.	Ref.	Ref.
Intermediate user	−0.14 (0.16); 0.40	−0.15 (0.16); 0.34	−0.14 (0.22); 0.53	−0.28 (0.22); 0.21
User	−0.33 (0.18); 0.07	−0.31 (0.18); 0.09	−0.04 (0.25); 0.87	−0.05 (0.25); 0.85
*Appendicular lean mass (%)*				
Nonuser	Ref.	Ref.	Ref.	Ref.
Intermediate user	−0.06 (0.17); 0.71	−0.08 (0.17); 0.65	−0.04 (0.23); 0.85	−0.18 (0.23); 0.43
User	−0.23 (0.19); 0.23	−0.22 (0.20); 0.27	0.17 (0.25); 0.49	0.17 (0.25); 0.51

^∗^ACE-inhibitor, ACE-I; angiotensin receptor blocker, ARB; ^†^Model 1 adjusted for scanner serial number and baseline lean mass measure; ^‡^Model 2 further adjusted for age, neighborhood socioeconomic status, race/ethnicity, smoking, physical activity (baseline and year 3), systolic blood pressure, diastolic blood pressure, height, diabetes, polypharmacy, depressive symptoms, arthritis, cancer, cardiovascular disease, hormone replacement therapy use, general health, physical function, dietary energy, dietary protein, healthy eating index, and clinical trial arm(s). Multiple imputation was used to fill in missing values for covariates (see Methods for details and Table 1 footnote for missing data frequencies).
